# COVID-19 Associated Pulmonary Aspergillosis: Diagnostic Performance, Fungal Epidemiology and Antifungal Susceptibility

**DOI:** 10.3390/jof8020093

**Published:** 2022-01-18

**Authors:** Nina Lackner, Claudius Thomé, Dietmar Öfner, Michael Joannidis, Timo Mayerhöfer, Rohit Arora, Eldina Samardzic, Wilfried Posch, Robert Breitkopf, Cornelia Lass-Flörl

**Affiliations:** 1 Institute of Hygiene and Medical Microbiology, Medical University Innsbruck, A-6020 Innsbruck, Austria; nina.lackner@i-med.ac.at (N.L.); eldina.samardzic@i-med.ac.at (E.S.); wilfried.posch@i-med.ac.at (W.P.); 2 Department of Neurosurgery, Medical University Innsbruck, A-6020 Innsbruck, Austria; claudius.thome@i-med.ac.at; 3 Department of Visceral, Transplant and Thoracic Surgery, Medical University of Innsbruck, A-6020 Innsbruck, Austria; dietmar.oefner@i-med.ac.at; 4 Department of Internal Medicine, Division of Intensive Care and Emergency Medicine, Medical University Innsbruck, A-6020 Innsbruck, Austria; michael.joannidis@i-med.ac.at (M.J.); timo.mayerhoefer@i-med.ac.at (T.M.); 5 Department of Trauma Surgery and Sports Medicine, Medical University Innsbruck, A-6020 Innsbruck, Austria; rohit.arora@i-med.ac.at; 6 Department of Anaesthesia and Intensive Care, Medical University Innsbruck, A-6020 Innsbruck, Austria; robert.breitkopf@tirol-kliniken.at

**Keywords:** aspergillosis, COVID-19, coronavirus disease 2019 (COVID-19)-associated pulmonary aspergillosis (CAPA), antifungal susceptibility testing, fungal diagnosis

## Abstract

Coronavirus disease 2019 (COVID-19)-associated pulmonary aspergillosis (CAPA) raises concerns as to whether it contributes to an increased mortality. The incidence of CAPA varies widely within hospitals and countries, partly because of difficulties in obtaining a reliable diagnosis. We implemented a routine screening of respiratory specimens in COVID-19 ICU patients for *Aspergillus* species using culture and galactomannan (GM) detection from serum and/or bronchoalveolar lavages (BAL). Out of 329 ICU patients treated during March 2020 and April 2021, 23 (7%) suffered from CAPA, 13 of probable, and 10 of possible. In the majority of cases, culture, microscopy, and GM testing were in accordance with CAPA definition. However, we saw that the current definitions underscore to pay attention for fungal microscopy and GM detection in BALs, categorizing definitive CAPA diagnosis based on culture positive samples only. The spectrum of *Aspergillus* species involved *Aspergillus fumigatus*, followed by *Aspergillus flavus*, *Aspergillus niger*, and *Aspergillus nidulans.* We noticed changes in fungal epidemiology, but antifungal resistance was not an issue in our cohort. The study highlights that the diagnosis and incidence of CAPA is influenced by the application of laboratory-based diagnostic tests. Culture positivity as a single microbiological marker for probable definitions may overestimate CAPA cases and thus may trigger unnecessary antifungal treatment.

## 1. Introduction

Invasive aspergillosis is frequently recognized in immunocompromised hosts, such as transplant recipients and patients with hematologic malignancies, patients receiving long-term or high dose steroids or other immunosuppressant drugs [1]. Aspergillosis associated with severe influenza virus infection (influenza-associated aspergillosis, IAPA) has been increasingly detected during the 2009 influenza A (H1N1) pandemic [2]. A 7-year retrospective study from Belgium and the Netherlands reported 14% and 31% of immunocompetent and immunocompromised patients being affected [3], respectively. The outbreak of the current severe acute respiratory syndrome coronavirus 2 (SARS-CoV-2) pandemic led to an increase of intensive care patients with severe pulmonary disorders [4]. Thus, several reports of coronavirus disease 2019 (COVID-19)-associated pulmonary aspergillosis (CAPA) [5,6,7] have raised concerns that this superinfection contributes to an increased mortality [8,9]. However, cases of CAPA were found to vary widely between hospitals and countries, intensive care units (ICU) reported rates from 3% to 33% [10]. These differences might be partly caused by difficulties in obtaining a reliable diagnosis and the lack of a specific clinical presentation; radiological features are not distinctive and diagnostic bronchoscopy is less frequently used in COVID-19 patients due to the risk of infectious aerosols [5]. In addition, a low sensitivity for circulating galactomannan (GM) in serum complicates any diagnostic evaluation, and the detection of *Aspergillus* species (sp.) in upper respiratory specimens does not distinguish fungal colonization from infection [5,10,11]. ECMM/ISHAM set consensus criteria for research and clinical guidance for defining and managing CAPA [5]. Here, CAPA is defined as possible, probable, or proven, based on host factors (patient with COVID-19 and requiring ICU care), clinical factors, and mycological evidence. During the first wave of the pandemic in Austria, we observed a slight increase of culture positive specimens (*Aspergillus* sp.) in the diagnostic laboratory of the Institute of Hygiene and Medical Microbiology, Medical University of Innsbruck, Innsbruck, Austria (unpublished data). To improve patient management, a routine screening of respiratory specimens (COVID-19 ICU patients) for *Aspergillus* sp. using culture and GM assays from serum or bronchoalveolar lavages (BAL) was implemented. We aimed to explore and describe our experience in diagnosing CAPA according to the newly defined consensus diagnostic criteria.

## 2. Materials and Methods

### 2.1. Collection of Respiratory Samples and Fungal Diagnostics

Cases of CAPA were identified during March 2020–April 2021 at the Medical University Hospital of Innsbruck (approximately 1600 beds, one of the biggest hospitals in Austria) by review of microbiological and infectious disease consultant data, with approval of the institutional review board. An intensive diagnostic screening procedure for *Aspergillus* sp. was implemented for all SARS-CoV-2 positive ICU patients. This included the usage of an additional *Sabouraud*-Glucose-*Agar* (SAB) plate for all routinely taken respiratory specimens. The incubation was done at 37 °C for 3 days; growth of *Aspergillus* sp. prompted species identification (MALTI-TOF MS) and the performance of GM detection (PlateliaAspergillus Test, Biorad, Vienna, Austria) in serum and/or respiratory fluids (tracheobronchial aspirates, BALs) and microscopy (Calcoflour white staining); screening independent fungal diagnostics were requested following the suspicion of fungal infections being present. The latter included GM detection from serum and/or BAL, and/or *Aspergillus* and panfungal PCR [12], microscopy, and culture. The standard of care clinical diagnostic procedures (e.g., chest radiograph or computer tomography scans (CT)) were routinely performed. Charts were reviewed to summarize demographic, clinical, and outcome data; cases were defined by CAPA criteria released in 2021 [5] by usage of pseudo-anonymized case report forms; another focus of interest was the epidemiology of *Aspergillus* sp. detected and the antifungal susceptibility profile against caspofungin, amphotericin B, voriconazole, posaconazole, isavuconazole, and micafungin. Susceptibility testing was performed using Etest^®^ (BioMerieux, Marcy-l’Étoile, Paris, France), once growth of fungal culture was evident.

### 2.2. CAPA Definitions

The diagnosis of probable pulmonary CAPA requires a pulmonary infiltrate or nodules, preferably documented by chest CT, or cavitating infiltrate (not attributed to another cause), or both, combined with mycological criteria [5]. The latter include positive microscopy in BAL (indicating molds), or culture positive BALs; serum GM index >0.5 or BAL GM index ≥1.0 or ≥2 *Aspergillus* PCR tests in plasma, serum, or whole blood; or a single positive *Aspergillus* PCR in BAL (cycle threshold <36 or in plasma, serum, or whole blood, and a single positive in BAL (any threshold cycle)) [5].

Possible pulmonary CAPA requires pulmonary infiltrate or nodules, preferably documented by chest CT, or cavitating infiltrate (which is not attributed to another cause) in combination with mycological evidence (e.g., microscopy, culture, or galactomannan, alone or in combination) obtained by non-bronchoscopic lavage [5].

## 3. Results

**Figure 1:**
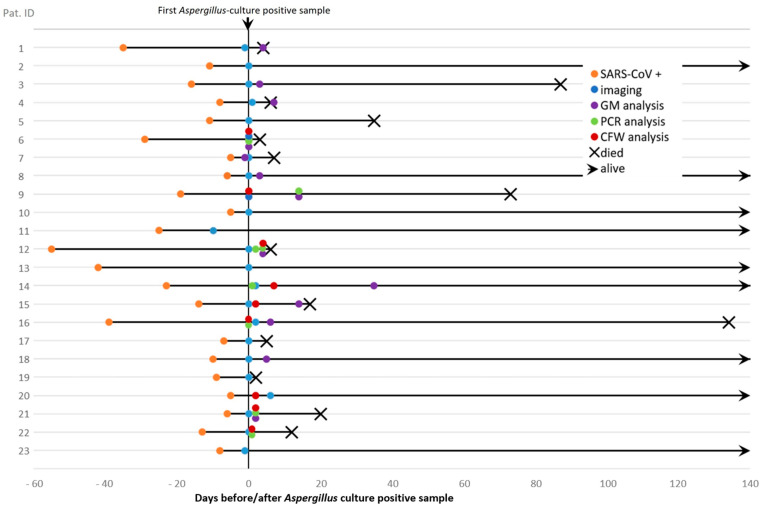
Timeline of patient cases calculated from the day of the first *Aspergillus* positive sample.

Our retrospective case study included 329 ICU patients diagnosed with SARS-CoV-2 included in the COVID-19 ICU registry [13], 23 of these patients (7%) revealed *Aspergillus* positive culture during hospitalization at the University Hospital Innsbruck, Innsbruck Austria. A total of 10 and 13 patients were classified to suffer from probable and possible CAPA. Microbiological details of culture, microscopy and GM testing are given in Table 1 and Table 2. In total, the study cohort included 6 female and 17 male *Aspergillus* culture positive individuals, aged between 39 and 84 years (median = 63 years), see Table 1. Duration of ICU stay ranged from 1 to 15 weeks and mostly implicated invasive mechanical ventilation. Underlying risk factors were diabetes mellitus (*n* = 10), being a regular (ex-)smoker (*n* = 8), severe obesity (*n* = 6), and organ transplantation (*n* = 6). Further, patients suffered from asthma, chronic obstructive pulmonary diseases and malignancies (Table 1). The administration of corticosteroids was documented for most, but not all patients. COVID-19 treatment followed according to the Austrian ICU SARS-CoV-2 treatment recommendations [14]. Patients were tested positive for SARS-CoV-2 5 to 55 days (median, 12 days) before proof of *Aspergillus* by culture, see Figure 1. All patients received broad-spectrum antibiotics and showed pulmonary infiltrates on imaging at the time-point of positive *Aspergillus* culture, herewith fulfilling CAPA entry criteria [5]. Antifungals initiated were voriconazole, posaconazole, amphotericin B, and anidulafungin or caspofungin, for drug regiments see Table 1. Antifungal treatment was done according to clinical guidance management of CAPA [5]; treatment duration varied form patient to patient and was continued either until death or for three more weeks. In particular, single patients received various combinations of antifungals, four patients classified as possible CAPA did not receive any antifungal treatment because of possible CAPA, and one patient was under fluconazole treatment due to severe *Candida* colonization of the respiratory tract, see Table 1 and Table 2. Fungal diagnostics were specifically requested for only three SARS-CoV-2 patients, see Table 2. Mortality was 67% and 42% for probable and possible CAPA.

**Figure 2:**
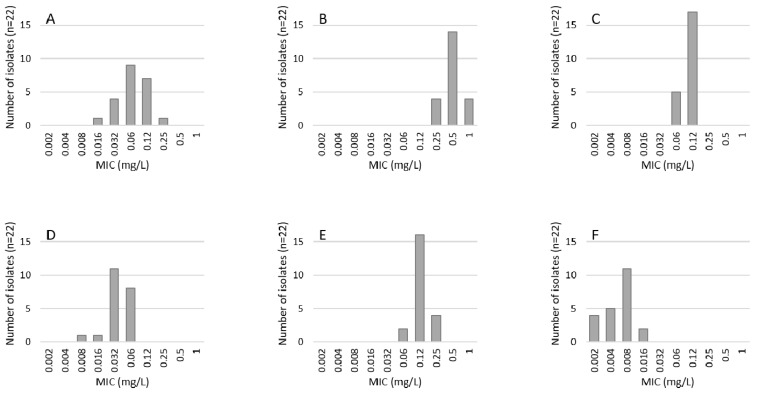
Distribution of minimal inhibitory concentrations (MIC) of (**A**) caspofungin, (**B**) amphotericin-B, (**C**) voriconazol, (**D**) posaconazol, (**E**) isavuconazol, and (**F**) micafungin determined by Etest^®^ of 22 *A. fumigatus* isolates from respiratory samples of COVID patients.

Species involved were *Aspergillus fumigatus* (*A. fumigatus*) (22), *Aspergillus flavus* (*A. flavus*) (2), and one case each of *Aspergillus nidulans* (*A. nidulans*) and *Aspergillus niger* (*A. niger*). Minimal inhibitory concentrations (MIC) for caspofungin, amphotericin B, voriconazole, posaconazole, isavuconalzole, and micafungin are presented in Table 2; only wild type strains for *A. fumigatus* were detected, see Figure 2.

## 4. Discussion

In this study, using the CAPA definition criteria proposed by Koehler et al. [5], we diagnosed 10 and 13 probable and possible cases, resulting in an incidence of 7% for CAPA in COVID-19 ICU patients in the University Hospital Innsbruck, Innsbruck, Austria. Interestingly, targeted fungal diagnostic tests were only requested for three ICU patients, suggesting that there was no clinical suspicion of an *Aspergillus*-related infection in the remaining 20 SARS-CoV-2 infected individuals. This highlights that the diagnosis and incidence of CAPA are strongly influenced by the application of laboratory-based diagnostic tests.

In addition, we underline difficulties in the assessment if culture positivity is equated with positive microscopy for probable definitions. A deeper analysis supports the differentiation between culture and microscopy reports, at least in SARS-CoV-2 ICU patients; relying on culture positivity without subsequent additional diagnostic work up may overestimate CAPA cases. We categorized 10 probable cases of CAPA, supported by either culture positive BAL, GM positive BAL/serum, or both (plus clinical factors being present), see Table 2. In most cases, culture microscopy and GM testing were in accordance. However, two patients (ID 3 and 9) with culture positive BAL had negative microscopy as well as negative GM tests in serum and BAL, respectively. GM detection in BAL is a valid test to confirm or rule out pulmonary aspergillosis with a sensitivity and specificity of both approximately 90% using an optical density cut-off of ≥1 in neutropenic patients [1]. It is well known that serum GM testing in SARS-CoV-2 patients is less accurate and that negative serum GM tests do not fully exclude an invasive fungal infection [5,15]. Apart from that, we observed that only patients with BAL positive microscopy presented with positive serum GM, suggesting a strong potential of microscopy in CAPA diagnosis. These patients could indeed represent “invasive” CAPA cases. Based on our results, we raise the question whether BAL culture positive but microscopic and GM negative cases suffer from possible rather than probable CAPA. We recommend a combination of fungal microscopy and GM testing in *Aspergillus* culture positive BALs for definitive diagnosis, being aware of the limited number of patients investigated. Another two patients (ID 4 and 7) had *Aspergillus* positive tracheal-secretions and positive GM borderline values (serum GM: 0.6) and hence were classified as probable CAPA cases; from a clinical point of view these patients had an infaust prognosis. Multiple serum GM testing is recommended to confirm or exclude the GM cut-off being exceeded or not. In general, culture positive tracheal-secretions and sputa led to possible CAPA definitions in our study population. The application of GM in serum was helpful for clinical decision making, as screening was negative in all cases. Based on clinical presentations, clinicians judged these patients as colonized rather than infected. Three possible CAPA cases underwent BAL, but microscopy and GM remained negative throughout hospitalization. Cut-off values for serum GM and BAL GM need specification for SARS-CoV-2 patients.

Mortality was high in patients with probable CAPA (67%) and possible CAPA (42%). These data are in agreement with several other studies [16,17] and underline the difficulties in the management of COVID-19 and superinfections in severe/critically ill patients. Demographic data and comorbidities were typical in our cohort and are in-line with other reports [5].

The spectrum of *Aspergillus* sp. involved was surprising for us as historically 30% of patients suffer from *Aspergillus terreus (A. terreus*) infection/colonization in Tyrol [18]. In this SARS-CoV-2 cohort, no single case of *A. terreus* was identified, instead *A. fumigatus*, followed by *A. flavus*, *A. niger*, and *A. nidulans* were detected. This is a rather unusual fungal epidemiology for the state Tyrol, Austria [19]. One reason could be that the study was restricted to the Innsbruck University Hospital, while the highest *A. terreus* incidences are found in the lower parts of Tyrol [20]. Otherwise, *A. fumigatus* is the most common species causing co-infections in COVID-19 patients [5]. The detection of *A. flavus* and *A. niger* from the upper respiratory tract secretions supports colonization rather than pulmonary infection [12].

Using Etest©, we did not detect azole resistance or other non-wildtype strains in *A. fumigatus. A. niger* and *A. flavus* showed higher MICs for amphotericin B and were above the EUCAST epidemiological cut offs (ECOFFs) of 4 and 0.5 mg/L, respectively [21]. This finding is not unusual as Gheith et al. (2014) found 67% of *A. flavus* to be above the ECOFFs for amphotericin B [22]. In addition, Taghizadeh-Armaki et al. (2017) report of a reduced susceptibility of *A. flavus* to amphotericin B [23]. Importantly, for *A. niger*, the ECOFF definition by EUCAST is still tentative [21] and the ECOFF by CLSI is 2 mg/L [24]. The recommended first-line therapy for CAPA is voriconazole or isavuconazole [5], azole resistance was not detected and clinical breakpoints for amphotericin B are currently under investigation. Others, however, report from the recovery of azole-resistant strains in COVID-19 ICU patients [25].

We are aware that our study suffers from several limitations such as a low number of BAL positive culture patients, retrospective nature, and only few patients with probable CAPA. Whether any *Aspergillus* colonization (surrogate marker) requires antifungal treatment needs further clinical evaluation.

## 5. Conclusions

The implementation of an additional SAB agar plate for respiratory tract secretions of SARS-CoV-2 patients in the routine led to an increase of *Aspergillus* positive cultures. In general, fungal reports supported a definitive CAPA diagnosis when culture, microscopy, and GM testing were in accordance. However, we emphasize that we should have paid more attention to microscopy and GM detection in BALs before categorizing definitive CAPA diagnosis; culture positivity as a single microbiological marker for probable definitions may overestimate CAPA cases. The shift from *A. terreus* to *A. fumigatus* in SARS-CoV-2 patients displayed a changing face of fungal epidemiology in the state of Tyrol, Austria, whereas antifungal resistance in vitro was not an issue.

## Figures and Tables

**Table 1:** Clinical data of 23 CAPA patients, defined according to ECMM/ISHAM criteria.

ID	Age (Y)	Sex (F/M)	Reason for Hospitalization	ICU Stay (Weeks)	IV (Weeks)	Underlying Risk Factors	Cortisteroid Treatment	CF and I	Antifungal Treatment	Death within 6 Weeks (Days after Culture Positivity, Cause)	CAPA
1	73	m	COVID-19	3–4	3–4	KTR, AVR	Yes	Yes	Ani	(4, COVID-19)	Probable
2	70	m	COVID-19	3–4	≤2	none	Yes	Yes			Possible
3	20	m	COVID-19	>4	>4	OB	Yes	Yes	Cas		Probable
4	84	m	COVID-19	≤2	≤2	DM, M	Yes	Yes		(6, Lung failure)	Probable
5	79	f	COVID-19	>4	>4	A	Yes	Yes	Amp	(35, COVID-19)	Possible
6	76	f	COVID-19, SMI	3–4	≤2	S		Yes	Amp	(3, Pneumonia)	Probable
7	77	f	COVID-19	≤2	≤2	DM, OB	Yes	Yes	Amp	(7, COVID-19)	Probable
8	56	m	COVID-19	>4	>4	COPD, DM, S, OB	Yes	Yes	Vor, Amp		Possible
9	61	m	COVID-19	>4	>4	none		Yes	Vor, Pos		Probable
10	50	m	COVID-19	≤2	≤2	DM	Yes	Yes	Amp		Possible
11	83	f	COVID-19	≤2	≤2	COPD, S, DM	Yes	Yes			Possible
12	39	f	COVID-19, cholecystectomy	≤2	≤2	KTR, OFD1		Yes	Ani, Amp	(6, Sepsis with MOF)	Probable
13	60	m	COVID-19	>4	>4	OB	Yes	Yes			Possible
14	59	m	UTI, COVID-19	>4	>4	DM, S, KTR, PTR, ex-TBP	Yes	Yes	Vor, Amp, Ani		Probable
15	53	m	COVID-19	3–4	≤2	none		Yes		(17, COVID-19)	Possible
16	58	m	CP, sepsis, COVID-19	>4	3–4	DM, NTR	Yes	Yes	Vor		Probable
17	82	m	COVID-19	≤2	≤2	DM, S		Yes	Vor, Amp	(5, MOF after COVID-19)	Possible
18	55	m	COVID-19	3–4	≤2	DM, OB	Yes	Yes	Vor, Amp		Possible
19	74	f	COVID-19	≤2	≤2	COPD, DM, S	Yes	Yes	Flu	(2, AKF after COVID-19)	Possible
20	47	m	COVID-19	>4	≤2	OB	Yes	Yes	Vor		Possible
21	72	m	COVID-19	3–4	3–4	M	Yes	Yes	Isa	(20, Lung failure)	Probable
22	75	m	COVID-19	3–4	3–4	S		Yes	Vor	(12, Sepsis with MOF)	Possible
23	83	m	COVID-19	3–4	≤2	S		Yes			Possible

Y: years; CF: clinical factors; I: imaging; CAPA: Coronavirus disease 2019 (COVID-19)-associated pulmonary aspergillosis; SMI: subacute myocardial infarction; UTI: urinary tract infection; CP: colon perforation; ICU: intensive care unit; IV: invasive ventilation; A: asthma; AVR: aortic valve replacement; COPD: chronic obstructive pulmonary disease; DM: diabetes mellitus; M: malignancy; S: (ex-)smoker; OB: obesity; KTR: Kidney transplantation; OFD1: orofaciales digitales syndrome type 1; PTR: pancreatic transplant recipient; ex-TBP: former tuberculosis patient; Ani: Anidulafungin; Cas: Caspofungin; Amp: Amphotericin B; Vor: Voriconazole; Pos: Posaconazole; Flu: Fluconazole; Isa: Isavuconazole; MOF: multiorgan failure; AKF: acute kidney failure.

**Table 2:** Microbiological data of 23 CAPA patients, defined according to ECMM/ISHAM criteria.

ID	Culture		Etest© MICs (mg/L)						GM		PCR		CFW		Fungal Diagnostics Requested by Clinician	CAPA
	SM	Species	CAS	AMB	VOR	POS	ISA	MICA	SM	Index	SM	Species	SM	Septate Mycelium		
1	TS	*A. fumigatus*	0.06	0.5	0.12	0.032	0.12	0.008	Serum	2.59	-	-	TS	Pos		Probable Possible
		*A. flavus*	0.008	8	0.12	0.12	0.12	0.002								
2	TS	*A. fumigatus*	0.06	0.5	0.12	0.032	0.12	0.008	Serum	Neg	-	-	-	-		Probable Probable
		*A. flavus*	0.016	4	0.12	0.12	0.12	0.002								
3	BAL	*A. fumigatus*	0.06	0.5	0.06	0.03	0.12	0.008	Serum	Neg	BAL	Neg	BAL	Neg		Possible
4	TS	*A. fumigatus*	0.12	0.5	0.12	0.06	0.12	0.008	Serum	0.63	-	-	-	-		Probable
5	TS	*A. fumigatus*	0.032	0.25	0.12	0.032	0.12	0.008	BAL	Neg	-	-	BAL	Neg		Probable
6	BAL	*A. fumigatus*	0.06	0.25	0.06	0.008	0.12	0.008	BAL	3.51	BAL	*A. fumigatus*	BAL	Pos	Culture, GM, PCR, CFW	Possible
7	TS	*A. fumigatus*	0.06	1	0.12	0.016	0.06	0.004	Serum	0.66	-	-	-	-		Probable
8	TS	*A. fumigatus*	0.06	0.25	0.12	0.032	0.12	0.008	Serum BAL	Neg	-	-	BAL	Neg		Possible
9	BAL	*A. fumigatus*	0.016	0.5	0.06	0.032	0.25	0.008	BAL	Neg	BAL	Neg	BAL	Neg	Culture, CFW	Possible
10	TS	*A. fumigatus*	0.06	0.5	0.12	0.06	0.12	0.004	Serum	Neg	-	-	-	-		Probable
11	SP	*A. fumigatus*	0.12	0.25	0.12	0.032	0.06	0.008	Serum	Neg	-	-	-	-		Possible
12	TS	*A. fumigatus*	0.12	1	0.12	0.06	0.12	0.004	BAL	4.83	BAL	*A. fumigatus*	BAL	Pos		Probable Possible
											EDTA-blood	*A. fumigatus*				
13	TS	*A. fumigatus*	0.25	0.5	0.12	0.06	0.12	0.004	Serum	Neg	-	*-*	-	-		Probable
14	TS	*A. fumigatus*	0.032	0.5	0.12	0.032	0.25	0.002	BAL	Neg	EDTA-blood	*A. fumigatus*	BAL	Pos		Possible Possible
		*A. nidulans*	0.032	0.25	0.06	0.06	0.032	0.004								
15	TS	*A. fumigatus*	0.12	0.5	0.12	0.032	0.12	0.004	Serum	Neg	-	*-*	BAL	Neg		Possible
16	BAL	*A. fumigatus*	0.12	0.5	0.12	0.06	0.25	0.008	BAL	6.82	BAL	*A. fumigatus*	BAL	Pos		Possible
17	TS	*A. fumigatus*	0.12	1	0.06	0.032	0.12	0.002	Serum	Neg	-	*-*	-	-		Probable
18	TS	*A. fumigatus*	0.12	1	0.12	0.032	0.12	0.016	Serum	Neg	-	*-*	-	-		Possible
19	TS	*A. fumigatus*	0.032	0.5	0.12	0.06	0.25	0.002	Serum	Neg	-	*-*	-	-		Possible
20	TS	*A. fumigatus*	0.03	0.5	0.06	0.03	0.12	0.008	Serum	Neg	-	*-*	BS	Neg		Probable
21	TS	*A. fumigatus*	0.06	0.5	0.12	0.06	0.12	0.016	BAL	6.69	BAL	*A. fumigatus*	BAL	Pos	Culture	Possible Probable
	BAL														Cult., GM, PCR, CFW	
22	TS	*A. fumigatus*	0.06	0.5	0.12	0.06	0.12	0.002	Serum	Neg	BS	Neg	BS	Neg		Probable
23	SP	*A. niger*	0.06	2	1	0.5	0.25	0.004	Serum	Neg	-	-	-	-		Possible

SM: sample material; TS: tracheal secretion; BS: bronchial secretion; BAL: broncho-alveolar lavage; SP: sputum; MIC: minimal inhibitory concentration; CAS: caspofungin; AMB: amphotericin B; VOR: voriconazole; POS: posaconazole; ISA: isavuconalzole; MICA: micafungin; GM: galactomannan detection; PCR: Polymerase Chains Reaction, targeting *Aspergillus*, CFW: Calcofluor white staining.

## Data Availability

Data will be made available by the corresponding author on request as far as data protection of the patients can be warranted.
